# A Systematic Treatise, Historical, Etiological and Practical, on the Principal Diseases of the Interior Valley of North America, as They Appear in the Caucasian, African, Indian and Esquimaux Varieties of Its Population

**Published:** 1851-04

**Authors:** 


*A Systematic Treatise, Historical, Etiological and Practical, on the Principal Diseases of the Interior Valley of North America, as they appear in the Caucasian, African, Indian and Esquimaux Varieties of its Population.* By Daniel Drake, M. D.

The great defect in American works on practical medicine has hitherto been the meagreness of their pages in facts essentially and practically local. It is true that American Journals embody a vast amount of this sort of information: but in systematic treatises, the aim appears rather to have been to write such works as would answer as text books for learners, than to collect and arrange those new and interesting features developed by difference of climate, position, and modes of life, and obtained only after great research and indefatigable labor. And yet no one will hesitate in awarding the higher meed of praise to works of this latter kind. The first furnishes the reader with opinions and conclusions, frequently ably drawn and executed with great force: the second supplies him with facts from which he is enabled to reason for himself. To the development of these local facts, Dr. Drake has addressed himself with all the energy peculiar to his character, and the result has been the production of a work of the greatest practical value to the medical world.

The germ of this treatise, Dr. Drake tells us in his preface, was a pamphlet, entitled "Notices concerning Cincinnati," written more than forty years since; but he set himself seriously to the task of its preparation about nine years ago, and for this purpose undertook long journeys throughout the entire interior valley of North America, in order to acquire the facts he has recorded, either from personal observation, or intercourse with medical men resident in the neighborhood described.

In setting forth upon this scientific pilgrimage, the author says "that he endeavored to leave behind him all opinions but the single one, that he who would observe correctly must have no theories to maintain or destroy." The result of these observations has been the collection of a vast amount of precisely that sort of information most useful to the medical practitioner and man of science.

It is our purpose, on some future occasion, to notice this work more at large, and in the meantime we would earnestly recommend it to our readers, as a most valuable contribution to medical literature, and worthy of the high reputation of its author.

